# Emergency department reorganisation introducing increased autonomy: A mixed effects approach to evaluate the effects of a national policy

**DOI:** 10.1371/journal.pone.0283325

**Published:** 2023-03-23

**Authors:** Line Stjernholm Tipsmark, Børge Obel, Tommy Andersson, Rikke Søgaard

**Affiliations:** 1 DEFACTUM, Central Denmark Region, Aarhus N, Denmark; 2 Department of Public Health, Aarhus University, Aarhus C, Denmark; 3 DESIGN EM–Research Network for Organizational Design and Emergency Medicine, Aarhus V, Denmark; 4 Department of Management, Aarhus University, Aarhus V, Denmark; 5 Interdisciplinary Centre for Organizational Architecture, Aarhus University, Aarhus V, Aarhus, Denmark; 6 Regional Hospital West Jutland, Herning, Denmark; 7 Department of Clinical Medicine, University of Southern Denmark, Odense, Denmark; Aalborg University: Aalborg Universitet, DENMARK

## Abstract

**Background:**

In 2007, a Danish national policy to future-proof emergency department (ED) performance was launched. The policy included several recommendations for the management and organisation of care that essentially introduced greater ED autonomy. In this study, we evaluate the effects of increased ED autonomy on readmission, mortality and episode costs for two large patient groups.

**Method:**

A non-randomised stepped wedge study-design where all EDs gradually implemented the policy at different steps during the study period (2008–2016). The timing and extent of policy implementation was determined from a retrospective cross-sectional survey of all 21 Danish EDs. This was linked to all episodes of hip fracture (n = 79,697) and erysipelas (n = 39,900) identified in the Nation Patient Registry and with episode-level outcomes. Mixed effect models were specified for the outcomes of 30-day readmission, 30-day mortality and episode costs, and adjusted for relevant ED- and episode-level heterogeneity.

**Results:**

Increased ED autonomy was associated with more readmissions (p<0.05) and higher episode costs (p<0.001) in hip fracture episodes. In erysipelas episodes, no general associations were found. When restricted to night-time admissions, increased ED autonomy was associated with poorer outcomes for erysipelas episodes and increased episode costs for both patient groups.

**Conclusion:**

The intended policy effects were not found for these two patient groups; in fact, reorganisation appeared to have harmed hip fracture patients and increased episode costs. Uncertainty remains regarding the longer-term consequences.

## 1. Introduction

The Emergency Departments (EDs) are essential to the care of acutely ill patients and the provision of healthcare system access. However, for many years EDs have become increasingly overcrowded due to multiple factors including population growth, increased complexity of care and limited resources [1–3]. ED crowding affects patient safety and has been described as a major healthcare issue affecting EDs worldwide [4]. In Denmark, EDs have been facing the same problems [1,2] and in 2007, the Danish Health Authority released a national policy of ED reorganisation [5]. The policy goals included consistent quality, continuity of care and efficient resource use [6]. The policy recommended several organisational changes that increase ED autonomy.

According to the national policy of ED reorganisation, the EDs should serve as the primary hospital entry, and some of the explicit recommendations therefore concerned clinical specialization, by anchoring case management in multidisciplinary teams and by making specialised equipment available at each ED. To further support patient diagnosing and flow, it was recommended to introduce triage and a flow coordinator function [2]. Senior physicians serving as frontline staff was the most ground-breaking feature of the new policy; before 2007 EDs were operated by junior physicians with limited opportunity to consult senior physicians [7]. In Denmark, a senior physician is a medical doctor, who has completed clinical training and a medical specialization. ED senior physicians could have a background in orthopaedics, internal medicine, general surgery or the like, since emergency medicine first was approved as a medical specialty in 2018. By directing, prioritizing and coordinating the ED tasks, senior physicians were meant to ensure a process-oriented workflow and organisational design at the ED. Most of the policy recommendations are premised on senior physicians being present at the ED.

Today, a decade after the national policy of ED reorganisation was introduced, the EDs have slowly developed increased autonomy [8]. Implementation of the recommended policies has come a long way, but substantial heterogeneity in ED autonomy prevails due mainly to the largely undefined terms and place of employment of senior physicians working at the ED [9,10].

Organisational design effects have been studied in hospital-based emergency care. A theoretical model widely used in the business literature, the multi-contingency model, was translated to match the ED setting by letting an expert panel select ED-relevant organisational design dimensions [10–13]. The multi-contingency model has been applied to Danish EDs and suggests that one dimension is affecting organisational performance: the degree of autonomy, which is closely linked to whether senior physicians are directly employed by the EDs as opposed to being on call from other departments [10,13].

In the present study we have chosen to use the multi-contingency model [14] to understand the organisational consequences of increased ED autonomy. The multi-contingency model assumes that work can be understood as information processing (IP). The model explains how organisational performance depends on a balance between the level of information an organisation needs to process to perform its tasks and the level of IP capacity an organisation possesses to handle the given information [15,16]. The fundamental premise is that increased uncertainty and complexity increases the demand for information processing. One of the main elements characterising an ED organisation is the complexity and uncertainty of its environment as patient visits are unscheduled and patients’ conditions are often unknown at arrival. Such complexity and uncertainty increase the IP needs; and the IP capacity must therefore be increased and/or the IP needs must be reduced. According to multi-contingency theory, we expect the IP capacity to increase, and IP needs to decrease if senior physicians are present at the ED [10].

Besides the availability of a validated model for characterising the ED organisations, the Danish context provides a natural experiment in that the implementation of the national policy of ED reorganisation has gradually led to increased ED autonomy [10,13]. It has previously been shown in one out of five Danish regions, that this movement is associated with improved 7-day mortality, but the wider effects remain uninformed; both in terms of generalisability to the entire system and in terms of other outcomes than mortality [13]. In this study, we take advantage of the natural experiment and evaluate the policy effect for the entire system on a broader palette of outcomes. Thus, the aim of this study was to evaluate the effects of increased ED autonomy on readmission, mortality and costs.

## 2. Materials and method

### 2.1 Setting

Denmark operates a primarily public, tax-financed healthcare system governed by five administrative regions [17]. In Denmark, around 40 public hospitals provide secondary healthcare, and 21 of these hospitals have EDs. The EDs serves as the primary hospital entry, and acutely ill patients account for approx. 71% of all inpatient and 8% of all outpatient admissions in Denmark [1,2].

### 2.2 Study design

In this observational study we used a non-randomised, stepped wedge study-design [18]. In our study, the 21 clusters (EDs) self-selected into the intervention (increased ED autonomy) at a time point of their own choice (stepwise implementation). Increased ED autonomy was based on internal employment of senior physicians at the ED. The timing of implementation was determined from a retrospective cross-sectional survey of all 21 Danish EDs by the end of the study period. All episodes at all Danish EDs were included between 1 January 2008 and 10 September 2016 and linked with the responsible ED.

### 2.3 Study populations

During the study period, the number of EDs decreased to 21. Thus, we selected the population based on diagnosis and not provider to maintain continuity in case mix over time. The study population was specified by all in- and outpatient emergency contacts of patients ≥18 years, registered with one of the International Classification of Disease version 10 (ICD-10) diagnoses; hip fracture (DS720, DS721, DS721A, DS721B and DS722) or erysipelas (DA469). These diagnoses were chosen by a chief physician responsible for one of the first-moving EDs with respect to reorganisation and discussed with the national DESIGN-EM research network (Research Network for Organisation Design and Emergency Medicine). Two criteria were used for initial selection: 1) high patient volume in order to ensure relevant statistical power and 2) therapeutical consistency over time as well as across EDs during the study period, in order to decrease the risk of confounding due to structural planning or introduction of new treatment options implemented during the study period. No formal statistical calculations were made with respect to power and it was considered irrelevant to generalise beyond the individual diagnoses. The choice of a surgical and a medical patient group characterised by a relatively stable context in terms of health care system structure and clinical guidelines was intended to serve as first cases. The patients were identified in Danish health registers.

The selection process and data massage are depicted in appendix S1 Fig Identification of the study populations.

### 2.4 Data

A combination of survey data on the EDs organisation and national register data on episodes was used. The survey was conducted in 2017 and targeted the management teams (chief physician and head nurse) at each ED. The key focus was whether and when they had initiated reorganisation of their department in line with the national policy. An ad hoc questionnaire was developed and electronically mailed to the management teams (S1 Questionnaire). Telephone follow-up, in case of non-response, was conducted and complete information was ultimately achieved for all EDs. The main respondent was the chief physicians at 16 EDs and the head nurse at 5 EDs, who altogether reported a mean employment period of 5.26 years (range 1–10 years).

National register data were retrieved from the Danish National Patient Register [19], the Danish Register of Causes of Death [20] and the Reference Cost database [21].

### 2.4.1 Outcomes

We used 30-day readmission to reflect quality. This was defined by acute readmission to any hospital department within 30 days after discharge in order not to underestimate readmission due to exemptions to the general admission structure where patients or their general practitioners were able to bypass the ED and contact the discharging department directly. Readmission included any readmission irrespective of geographical location because of the general prehospital practice where a patient is usually brought into the nearest ED. Readmission excluded contacts concerning cancer treatment, accidents and mental disease, in line with national monitoring guidelines [22]. We used 30-day mortality as a health measure, defined by mortality within 30 days after the day of diagnosis [23]. Admission costs were estimated in 2018-DKK (adjusted for inflation using the general consumer price index) and included resource use from admission to the ED through hospital discharge. Costs were log-transformed.

#### 2.4.2 Intervention

The selected interventions were based on themes from the Danish ED policy concerning increased ED autonomy [5,24]. Overall, the themes covered availability of senior physicians and different flow coordinating initiatives. According to previous studies senior physician employment, is the main driver of ED autonomy. Thus, senior physician employment was selected as our primary intervention for analyses, and it was defined as duration of senior physician´s employment at the ED (year) (S1 Table). Due to the protection of individual, authorised health care professionals it is not possible to analyse or report the actual years of seniority of individual employees. All selected interventions were included in the sensitivity analyses.

#### 2.4.3 Heterogeneity

Department heterogeneity was assessed in terms of teaching status, last year’s total episode volume and last years’ means for 30-day readmission, 30-day mortality and log (episode costs).

Gender, age and comorbidity were used to adjust for episode heterogeneity. We used the Elixhauser Comorbidity Index to define comorbidity [25]. The index was originally developed with 30 indicators, but we used the updated version with 31 indicators for the ICD-10 [26,27].

### 2.5 Statistical analyses

We analyze the stepped-wedge design by standard mixed effects regression for each of the patient populations (hip fracture and erysipelas) and for each individual outcome (readmission, mortality, and costs). Due to the scaling of the outcomes we used logistic for binary outcomes and linear for the continuous outcome. In the standard mixed effects regression, fixed effects are specified for the group-average structure and random effects for the heterogeneity [28]. By design, the assignment of intervention to clusters is monotone and confounded with time, because EDs crossover from control to intervention on a staggered schedule. We separate secular trends from the group-average intervention effect by inclusion of calendar time dummies [29]. The models were assessed by the Wald test and a 5% significance threshold. To avoid potentially inflated type 1 error, inclusion of 30 clusters or more is recommended [30]. Since the present analyses comprise only 21 clusters, small sample correction was applied using the t-distribution rather than the normal distribution to construct confidence intervals [31]. All variables included in the mixed effects models are shown in S1 Table.

In Table 1 we have chosen three years (2008, 2012, 2015) to describe the evolution of the population (on department and episode level) over time. The dates represent the start, middle and end of the period (whole calendar years).

**Table 1:** Characteristics of Danish emergency departments (m = 21) and the episodes they manage for two diagnostic populations at selected years.

	**2008**	**2012**	**2015**
**Hip fracture (n)**	**9,341**	**9,016**	**11,236**
Episode mean (SD)			
Male gender (%)	0.30 (0.46)	0.32 (0.47)	0.32 (0.47)
Age (years)	79.06 (12.10)	79.00 (12.06)	78.93 (12.32)
Elixhauser Index^a^	0.26 (0.61)	0.28 (0.66)	0.31 (0.72)
30-day readmission (%)	0.11 (0.31)	0.11 (0.31)	0.09 (0.29)
30-day mortality (%)	0.10 (0.30)	0.10 (0.30)	0.09 (0.28)
Episode cost (DKK 2018)	79,489 (65,486)	75,283 (63,325)	50,533 (55,209)
Departments mean (SD)			
Teaching status (%)	0.15 (0.37)	0.15 (0.37)	0.20 (0.41)
Episode volume	466 (243)	438 (226)	363 (189)
30-day readmission (%)	0.10 (0.08)	0,09 (0.03)	0.12 (0.04)
30-day mortality (%)	0.09 (0.02)	0.11 (0.04)	0.09 (0.02)
Episode cost (DKK 2018)	78,444 (26,283)	66,155 (32,594)	66,988 (23,646)
**Erysipelas (n)**	**3,224**	**4,338**	**6,433**
Episode mean (SD)			
Male gender (%)	0.56 (0.50)	0.56 (0.5)	0.58 (0.49)
Age (years)	62.73 (17.24)	63.52 (17.60)	60.84 (18.34)
Elixhauser Index^a^	0.48 (0.82)	0.43 (0.79)	0.31 (0.73)
30-day readmission (%)	0.13 (0.33)	0.13 (0.34)	0.14 (0.35)
30-day mortality (%)	0.02 (0.13)	0.02 (0.14)	0.02 (0.13)
Episode cost (DKK 2018)	32,842 (44,985)	26,800 (35,269)	20,020 (34,644)
Departments mean (SD)			
Teaching status (%)	0.14 (0.36)	0.15 (0.37)	0.20 (0.41)
Episode volume	140 (86)	196 (109)	305 (274)
30-day readmission (%)	0.11 (0.03)	0.12 (0.03)	0.15 (0.04)
30-day mortality (%)	0.02 (0.02)	0.02 (0.01)	0.02 (0.01)
Episode cost (DKK 2018)	35,113 (14,145)	29,839 (16,446)	24,890 (8,962)

ED = emergency department.

^a^ Total, unweighted score (the individual variables cannot be shown according to the General Data Protection act).

There were no missing data except for the lagged variables of mean episode costs at two departments where this information was missing for two and three periods, respectively. The missing values were imputed by the specific department’s means of earlier years. All analyses were conducted in Stata/MP version 15.1.

#### 2.5.1 Sensitivity analyses

In the first sensitivity analysis we included seven policy recommendations (whether: 1) senior physicians are employed at the ED, 2) senior physicians are available 24h, 3) senior physicians from other departments can be consulted when needed, 4) the ED activities are managed by flow coordinators 5) patients treatment are managed by multidisciplinary teams, 6) staff in the ED is able to make independent decisions concerning patient management without consulting physicians from other departments, and 7) ED facilities are located in one building) concerning increased ED autonomy [5,10], to test the interdependence of the recommendations. In the second sensitivity analysis we applied an interaction term of the ED autonomy and the time of patient episode (daytime 7:00 a.m.-10:59 p.m., night-time 11:00 p.m.- 6:59 a.m., matching employee work schedule). This was done to test if our models mask some effects of the increased ED autonomy, since day- and night-time effects are counteracting [10,12,32] In the third sensitivity analysis, patient-level analyses were performed instead of episode-level analyses to determine the consequences of between-episode dependence, meaning that a patient can die several times in our data. Hence, data were restricted to first-time episodes to test if this affected the 30-day mortality. Furthermore, we tested 7- and 30-day outcomes, since clinical consensus between 7- and 30-day outcomes is not established. Finally, the cost measure was extended to cover readmission costs for the time window of costs and outcomes to match.

## 3. Results

Despite reorganisation of the hospital-based emergency care, episode and department characteristics have remained largely stable over time for the chosen diagnoses (Table 1). During the study period, all episodes of hip fracture (n = 79,697) and erysipelas (n = 39,900) were included. From the episode characteristics, we observe that hip fracture episodes are endured primarily by women with a mean age of around 79 years, with 30-day readmission and 30-day mortality of roughly 10%. Episode costs appear to decrease in 2015, which can be partially explained by implant cost reductions. Department characteristics remain stable over time; yet episode volume seems to decrease over the selected years. Erysipelas episodes have an almost even gender distribution and appear at a mean age of around 62 years, with a 30-day readmission of approximately 13% and a 30-day mortality of 2%. We observe a potential decrease in episode costs in 2015. Department characteristics remain stable over time, though an increase in episode volume seems to occur.

**Fig 1:**
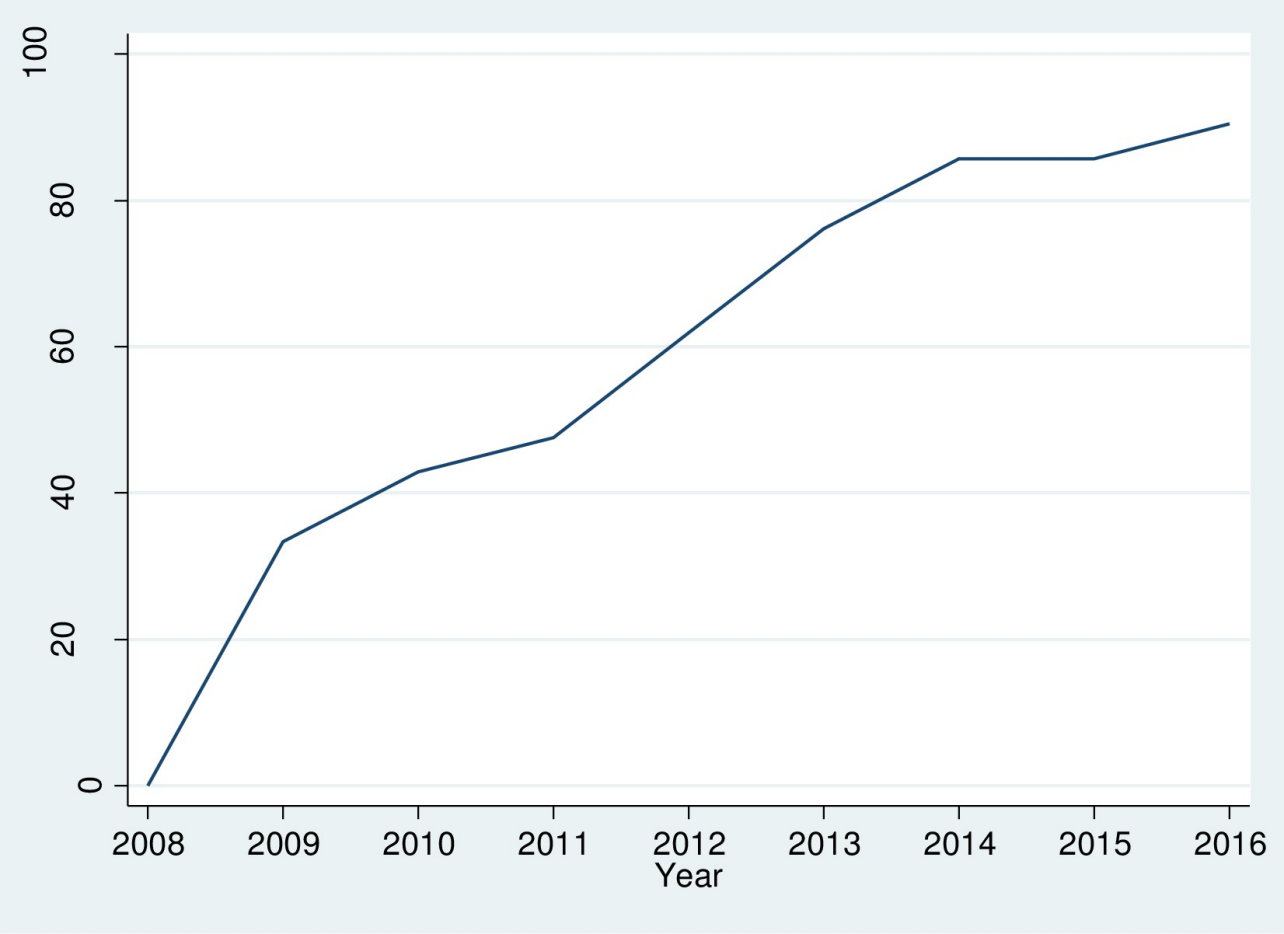
Implementation percentage (%) of increased emergency department autonomy over time at Danish emergency departments (N = 21). Increased autonomy is defined as senior physician employment at the emergency department.

From our survey data, we observe that the national policy recommendations for increased ED autonomy (Fig 1) have been implemented gradually during the study period (2008–16). Yet, no increase is observed between 2014 and 2015. Increased ED autonomy is reported for 90% of the Danish EDs (19 out of 21 EDs) in 2016.

Due to the set-up of the Reference Cost database, 11% of the data were missing (excluding 2016 data). Missing episode costs were not imputed. Unadjusted and adjusted mean outcomes over time for episodes managed by departments with versus departments without increased ED autonomy were used for graphical representation (S2A and S2B Fig).

In the main analyses, we find that the effect of increased ED autonomy primarily affects hip fracture patients, whereas results are insignificant for patients with erysipelas (Table 2). For patients with hip fracture, increased ED autonomy is associated with an increased readmission rate of 3% per year (p<0.05). This corresponds to an average increase from 889 readmissions for EDs without increased autonomy to 915 readmissions for EDs with increased autonomy. Episode costs increased by 6% per year (p<0.001) within EDs with increased autonomy. This corresponds to an increase from 73,471 DKK at EDs without increased autonomy to 77,771 DKK for EDs with increased autonomy. The unadjusted and adjusted mean association between duration of increased autonomy and outcomes can be found in S2C and S2D Fig.

**Table 2:** Effects of increased emergency department autonomy on mortality, readmission and admission costs based on mixed effect models.

	30-day readmission	30-day mortality	Episode costs
	OR (95% CI)	OR (95% CI)	Log cost (95% CI)
**Hip fracture**			
Increased autonomy	**1.030 (1.001–1.059)**	1.014 (0.985–1.044)	**0.059 (0.049–0.069)**
Model diagnostics			
n	79,079	79,072	64,763
Min episodes per ED	367	366	247
Max episodes per ED	7,646	7,645	7,033
**Erysipelas**			
Increased autonomy	0.996 (0.967–1.024)	1.046 (0.976–1.117)	-0.005 (-0.022–0.011)
Model diagnostics			
n	39,626	39,411	30,692
Min episodes per ED	122	122	122
Max episodes per ED	5,255	5,206	3,675

CI = confidence interval, ED = emergency department, OR = odds ratio.

Results are coefficients from mixed effects models expressing the effect of each additional year of senior physicians being employed by the emergency department; 30-day readmission and 30-day mortality are OR (95% CI) and episode costs are log cost (95% CI). All estimates are adjusted for all covariates shown in Table 1 (episode-level age, gender and comorbidity and department-level teaching status, episode volume, and average episode costs, 30-day readmission and 30-day mortality).

### 3.1 Sensitivity analyses

We tested the definition of increased autonomy (Table 3) including all interventions in our model. We observe that the explanatory power changes from senior physician employment to having decision authority at the ED.

**Table 3:** Results of sensitivity analyses based on alternative specification of emergency department autonomy.

	30-day readmission	30-day mortality	Episode costs
	OR (95% CI)	OR (95% CI)	Log cost (95% CI)
Hip fracture (n = 79,697)			
Senior physicians employed at the ED	1.004 (0.886–1.137)	1.004 (0.887–1.135)	**0.067 (0.028–0.105)**
Senior physicians 24-hour	0.944 (0.827–1.076)	1.120 (0.982–1.276)	**-0.113 (-0.154- -0.070)**
External senior physicians	1.035 (0.925–1.158)	0.960 (0.857–1.073)	**-0.220 (-0.256- -0.184)**
Flow coordinator	0.967 (0.859–1.089)	0.989 (0.876–1.114)	**0.113 (0.077–0.150)**
Multidisciplinary teams	1.049 (0.929–1.176)	1.077 (0.954–1.214)	**-0.070 (-0.105- -0.035)**
Decision authority	**1.138 (1.021–1.267)**	1.020 (0.913–1.137)	**0.056 (0.021–0.092)**
Facilities in one building	0.978 (0.865–1.104)	1.025 (0.907–1.158)	**0.086 (0.044–0.128)**
Erysipelas (n = 39,900)			
Senior physicians employed at the ED	**0.797 (0.685–0.927)**	1.380 (0.986–1.920)	**0.178 (0.105–0.251)**
Senior physicians 24-hour	1.004 (0.857–1.176)	0.882 (0.631–1.234)	**-0.284 (-0.365- -0.202)**
External senior physicians	0.974 (0.853–1.112)	**0.650 (0.477–0.883)**	**-0.221 (-0.290- -0.152)**
Flow coordinator	1.056 (0.910–1.225)	1.251 (0.885–1.762)	**0.094 (0.025–0.163)**
Multidisciplinary teams	**1.220 (1.051–1.415)**	1.150 (0.800–1.660)	-0.060 (-0.126–0.008)
Decision authority	0.928 (0.815–1.056)	0.764 (0.571–1.022)	**0.192 (0.118–0.265)**
Facilities in one building	0.935 (0.820–1.067)	1.140 (0.853–1.522)	**0.416 (0.332–0.498)**

OR odds ratio, CI confidence interval, ED emergency department.

Results are coefficients from mixed effects models expressing the effect of each additional year of senior physicians being employed by the emergency department; 30-day readmission and 30-day mortality are OR (95% CI) and episode costs are log cost (95% CI). All estimates are adjusted for all covariates shown in Table 1 (episode-level age, gender and comorbidity and department-level teaching status, episode volume, and average episode costs, 30-day readmission and 30-day mortality).

When including episode time (Table 4), we find that for patients with hip fracture night-time episodes are overall associated with increased readmission (p<0.05), mortality (p<0.05) and episode costs (p<0.001) compared with day-time episodes. A similar impact of night-time admissions is observed for patients with erysipelas, though the impact reached statistical significance only for episode cost (p<0.001). Changing from episode- to patient-level analyses was not associated with a change in 30-day mortality. Seven-day outcome measures changes readmission of patients with hip fracture from a significant to an insignificant increase. Inclusion of readmission costs did not change the results.

**Table 4:** Results of sensitivity analyses assessing alternative specification of outcomes.

	30-day readmission	30-day mortality	Episode costs
	OR (95% CI)	OR (95% CI)	Log cost (95% CI)
Hip fracture (n = 79,697)			
Interaction admission time of day			
07.00 am-10.59 pm	1.026 (0.996–1.055)	1,008 (0.980–1.036)	**0.052 (0.040–0.063)**
11.00 pm-06.59 am	**1.045 (1.012–1.079)**	**1.038 (1.004–1.072)**	**0.086 (0.074–0.097)**
First-time episodes (n = 65,209)		1.013 (0.982–1.044)	
7-day outcomes	1.020 (0.980–1.060)	1.024 (0.981–1.068)	
Episode costs + readmission costs			**0.061 (0.050–0.071)**
Erysipelas (n = 39,900)			
Interaction admission time of day			
07.00 am-10.59 pm	0.995 (0.967–1.023)	1.047 (0.982–1.115)	-0.012 (-0.025–0.004)
11.00 pm-06.59 am	1.001 (0.964–1.039)	1.039 (0.947–1.138)	**0.080 (0.057–0.100)**
First time episodes (n = 30,269)		1.048 (0.989–1.109)	
7-day outcomes	0.991 (0.955–1.028)	1.050 (0.953–1.156)	
Episode costs + readmission costs			-0.001 (-0.018–0.015)

OR odds ratio, CI confidence interval.

Results are coefficients from mixed effects models expressing the effect of each additional year of senior physicians being employed by the emergency department; 30-day readmission and 30-day mortality are OR (95% CI) and episode costs are log cost (95% CI). All estimates are adjusted for all covariates shown in Table 1 (episode-level age, gender and comorbidity and department-level teaching status, episode volume, and average episode costs, 30-day readmission and 30-day mortality).

## 4. Discussion

The overall results of the study indicate that increased autonomy have had no positive consequences related to readmission, mortality and episode cost for hip fracture and erysipelas episodes; in fact, the most consistent results throughout our main and sensitivity analyses was that the longer time with increased autonomy the higher readmission rated and episode costs (hip fracture only).

### 4.1. Generalizability of the effect of emergency department autonomy

Multi-contingency theory and a recent study support the potential advantage of increased ED autonomy [13,16,33]. The study analysed the effect of increased ED autonomy on ED discharge rates in one of the five regions in Denmark from 2011–2014. The study showed an odds ratio (OR) for death within 7 days of discharge of 0.72 (95% CI 0.59–0.92). For both our patient groups, we find opposite though statistically non-significant tendencies. Since the study did not include readmission and episode costs, we do not know if similarities exist in this respect. The study analysed the effect in one region, but we were able to include the whole nation. Moreover, different study designs, e.g. in terms of inclusion criteria and analyses, were applied, which together might explain the different findings. According to theory, performance will be affected if the IP capacity does not match the IP demands 24-hours a day. This theoretical perspective is supported by recent findings [32], where increased mortality was associated with ED patients being admitted at weekend evenings (OR 1.32; 1.03–1.70) and during night-time (OR 1.29; 0.90–1.84) compared with weekday daytime. Furthermore, the study investigated the causes of this increased mortality and concluded that changes in the ED organisational design caused considerable misfits [12]. They found that the changes among others encompassed exchange of ED physicians with fewer physicians from different departments (including only one ED physician). A lack of manpower, skills, hospital support services and flexibility, and thereby low IP capacity, heavily burdened physicians and forced them to have limited situation perspective which brought patients in harm’s way. The study states that the same issues can be found out-of-hours in weekdays, as also indicated by our results.

In theory, ED autonomy involves a transition from a functional- to a process-oriented workflow. A study investigated the relations between incentives and a process-oriented workflow (ED autonomy) in acute orthopaedic pathways (patients with hip fracture) [34]. A team of orthopaedists had regular shifts in a Danish ED; however, due to staff shortage in the department of orthopaedic surgery, junior physicians were taking many of these shifts. The study found a substantial difference in the number of doctors available (present or on-call) at the ED during evening/night time compared to daytime. This meant that patients were prioritised according to assigned level of acuteness of their condition, and patients with hip fracture were often not at the top of the list. These observations are supported by our sensitivity analysis where we found night-time episodes for hip fracture patients to be associated with increased readmission, mortality and costs. In addition, the orthopaedic surgeons felt frustrated about the working conditions and their lack of influence on these ED conditions. They therefore felt that they were not delivering the best quality of care to the patients with hip fracture, and they felt that their professional autonomy was violated. Furthermore, they had low intrinsic motivation to contribute to the hip fracture pathway, since these patients and the majority of the other patients with orthopaedic issues in the ED were rather trivial for orthopaedists. These results, together with the results of the present study, highlight the exceedingly difficult balance between the functionally oriented (non-ED autonomy) and the process-oriented workflow (ED autonomy) [34]. In the business literature, this is a well-known phenomenon that must be dealt with in order to secure that performance goals are reached [16,35]. If good communication and work relations between the ED and the remaining hospital is not established, this will most likely affect patient treatment and outcome.

### 4.2 Policy implications

Analysing ED effects is particularly difficult because for many patients, the ED is the first point of contact with the hospital during their admission. Separating the outcome of the quality of care provided at the ED from the outcome of any subsequent admission in other hospital departments is therefore difficult. Yet, the ED is not supposed to function as a 100% independent unit; hence, the results are likewise bound to reflect the cooperation between the ED and the rest of the hospital, as previously highlighted. This may explain the difference observed between the two patient groups in the present study. Patients with hip fracture demand highly specialised treatment that can be performed only by orthopaedic surgeons. Patients with erysipelas, on the other hand, demand a more universal treatment that can be provided by a broader range of staff [36]. Hip fracture outcome could therefore be more vulnerable to miscommunication and lack of collaboration with non-ED staff than erysipelas.

To maintain increased ED autonomy, 24-hour presence of senior physicians in the ED is essential [10]; however, primarily due to recruitment challenges this was not possible at all ED [2,24,37]. Emergency medicine was only approved as a medical specialty in Denmark in 2018, so it will take a while to recruit the needed workforce of fully-trained senior physician in emergency medicine [38].

A relevant consideration in relation to the meaning of the study is the experience of senior physicians, which is the actual exposure we assess the outcome of. Due to Danish regulation for the protection of individual physicians’ anonymity, it is not possible to quantify the actual number of years of experiences, and exposure is defined according to specialised versus non-specialised (specialised refers to physicians having undergone 5–6 years of formal supervised clinical experience after their authorisation). This definition is highly relevant in the Danish system in that historically, only non-specialised, junior physicians served as frontline staff in the EDs, and the national policy overall intended to bring seniors physicians at the frontline. This was a major organisational change. Once the reorganisation is fully implemented, it will be relevant–as a next step–to examine the effects of more experienced seniors versus less experienced seniors.

### 4.3 Recommendations

A priority must be to support complete policy implementation and secure sustainable co-operation agreements between the EDs and the remaining hospital departments. Further research is needed to fully understand the long-term effects of full-scale ED autonomy, and a broader range of patient groups must be included to grasp the effect of increased ED autonomy.

### 4.4 Strength and weaknesses

In the field of ED organisation, the present study is unique owing to the size of its study population and the length of the study period. Still, using an extensive study period can complicate the overview of initiatives affecting these patient groups. For example, during the study period, complexity of patients presenting to EDs has increased, ED overcrowding has worsened and numerous care models of have been introduced. For instance, a policy of emergency teams was introduced [39]. These teams can manage some non-complex emergency patients in their own home. We expect the effect to be minimal, since many of the study patients did not belong to the emergency teams’ main target group and we account for patient comorbidity. While we used a fairly robust design and adjusted for a number of prognostic and organisational variables, residual confounding due to these issues cannot be ruled out.

Another methodological consideration is our use of a numerical definition of exposure as opposed to a dummy definition. We know from the literature that reorganisation does not happen overnight or even over a specific year [2,24,37] and we thus reflect a dose-response relationship where the number of years since an ED management decided to adapt the national policy about bringing seniors in front matters. This is however not without assumptions in that we do not know exactly how many were hired when but instead assume a linear implementation. If the implementation was instead skewed to early or late actual overweight of seniors in front, we risk over- or underestimating the effect nut, again, previous studies on implementation describe a largely linear scheme [2,24,37].

A study strength is the complete collection of survey data. Nevertheless, the depth of these data is limited. From the survey we only know when the EDs started to hire senior physicians; we have no information on the number of senior physicians employed at the individual ED, and how this changed over time. This dichotomisation of the real world could potentially underestimate the results. In addition, selection bias might be a problem in the survey data used to define increased ED autonomy. Since the Danish EDs have been under extraordinary pressure during this transition period, staff turnover has been high. Hence, the respondent might not have been employed during the whole reorganisation period, potentially affecting the data quality.

The cost perspective focuses on episode costs. This can be perceived as both a strength and a limitation. The strength lies in the specific focus on ED services, and a limitation lies in the lack of measures capturing the societal effect of the policy. However, a major strength is the data upon which the cost perspective is based. The Reference Cost database provides the number of available tariffs and thereby the actual variation in episode costs as opposed to the diagnosis-related grouping (DRG) tariff, which is based on national averages. Unlike the rest of our register data, this unique Reference Cost database unfortunately comes at the price of missing data. The mixed effect models applied in our study are fit to handle missing data [40,41]. We do not expect the missing data to be connected to department performance, since missing data is a matter of reporting accounts. We imputed missing department cost when it was possible to retrieve information from the previous year.

Our study was designed as a stepped-wedge study which has some advantages and disadvantages. Evaluation of health policies depends on already defined settings; hence, the often suboptimal setup places high demands on the choice of analyses. On average, in our study, the intervention condition is later in time than the control condition. The intervention effect is therefore confounded by underlying temporal trends. The stepped-wedge design takes this factor into account, thereby avoiding a biased intervention effect. On the downside, the small number of clusters in our study [21] could inflate type 1 error, and small-sample corrections were applied.

The analyses are based on several definitions and assumptions that could affect the results. To test the study assumptions, sensitivity analyses were performed. They showed overall robust results. Autonomy was based on the duration of senior physicians’ employment at the ED since literature supports this assumption, and our data show a high correlation between senior physician employment and several reorganisation elements; having multidisciplinary teams, flow coordination, senior physicians available 24-hours and the ability to make independent decisions concerning patient management without consulting physicians from other departments, we find this assumption reasonable.

### 4.5 Conclusion

The intended policy effects from reorganisation of Danish EDs were not found. On the contrary, it appeared that reorganisation could have harmed patients and increased episode costs for one large patient group. Uncertainty remains regarding the complex and possibly mediating role of implementation as well as the longer-term consequences. Our previous studies on implementation highlighted communication and collaboration issues that could worsen, at least temporarily, by reorganisation. Finally, the observation of poorer outcomes during night-times underlines the importance of 24-hour coverage by the quality-supporting initiatives such as senior physician coverage.

## Supporting information

S1 Fig Identification of the study populations. ED = emergency department.
(TIF)

S2 Fig A. Unadjusted outcomes over time for episodes managed by departments with versus departments without increased autonomy. Note: Values are yearly means across departments. B. Adjusted outcomes over time for episodes managed by departments with versus departments without increased autonomy. Note: Values are yearly means across departments. Adjustment is based on the mixed effects models of the main analysis, which includes all variables shown in manuscript Table 2. C. Unadjusted outcomes over the duration of time with increased autonomy (time since implementation). Note: Values are yearly means across departments. D. Adjusted outcomes over the duration of time with increased autonomy (time since implementation). Note: Values are yearly means across departments. Adjustment is based on the mixed effects models of the main analysis, which includes all variables shown in manuscript Table 2.
(TIF)

S1 Table Variables included in the mixed effects models. ED = Emergency department, EPI = episode, DPT = Department.
(DOCX)

S1 Questionnaire (DOCX)
