# Supplementary figures and images for: Emergency department reorganisation introducing increased autonomy: A mixed effects approach to evaluate the effects of a national policy
Source: PLoS One. 2023 Mar 23;18(3):e0283325. doi: 10.1371/journal.pone.0283325 (PMC10035920; doi:10.1371/journal.pone.0283325)

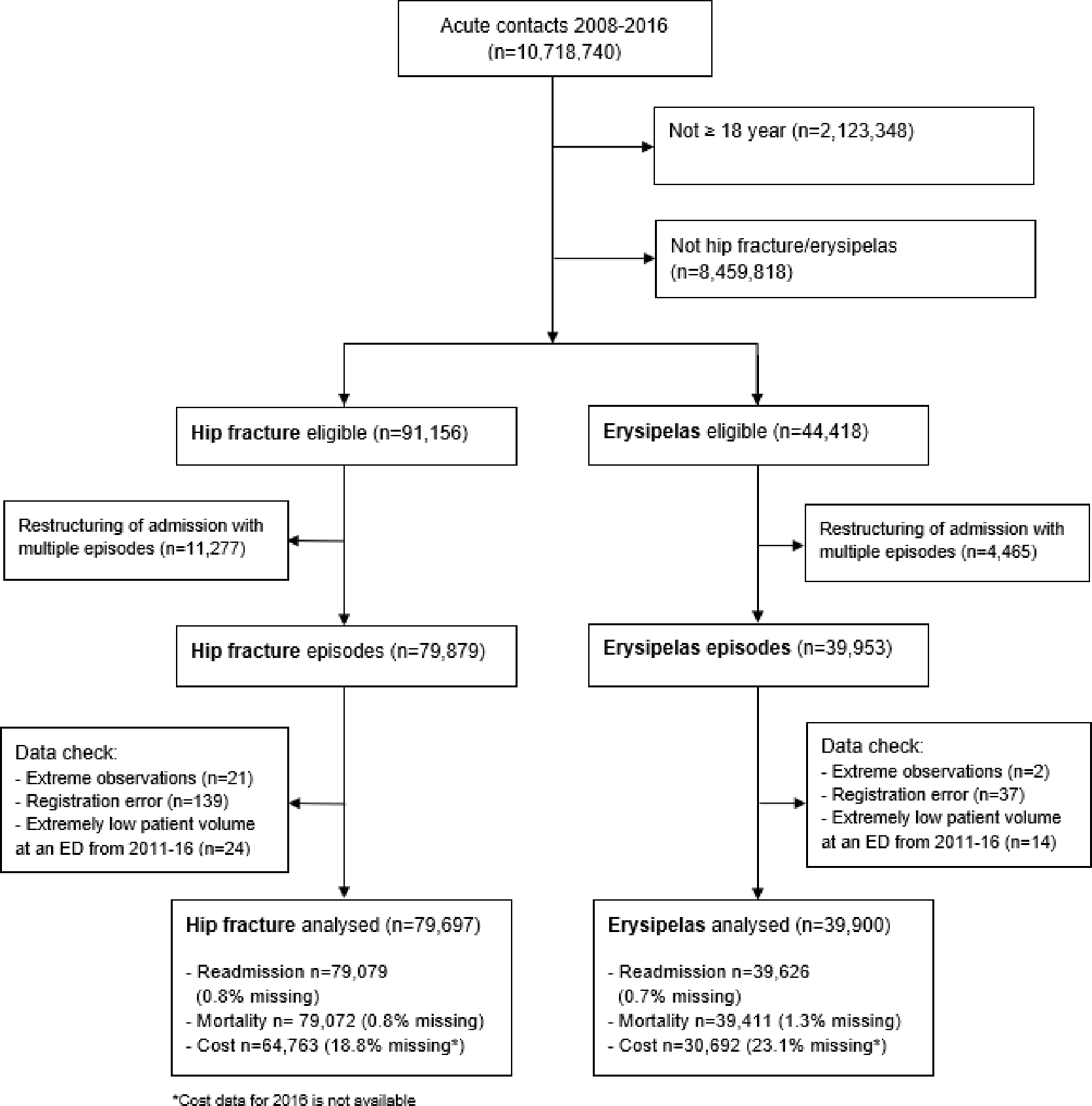

Supplement: S1 Fig — ED = emergency department. (TIF) [file pone.0283325.s001.tif]

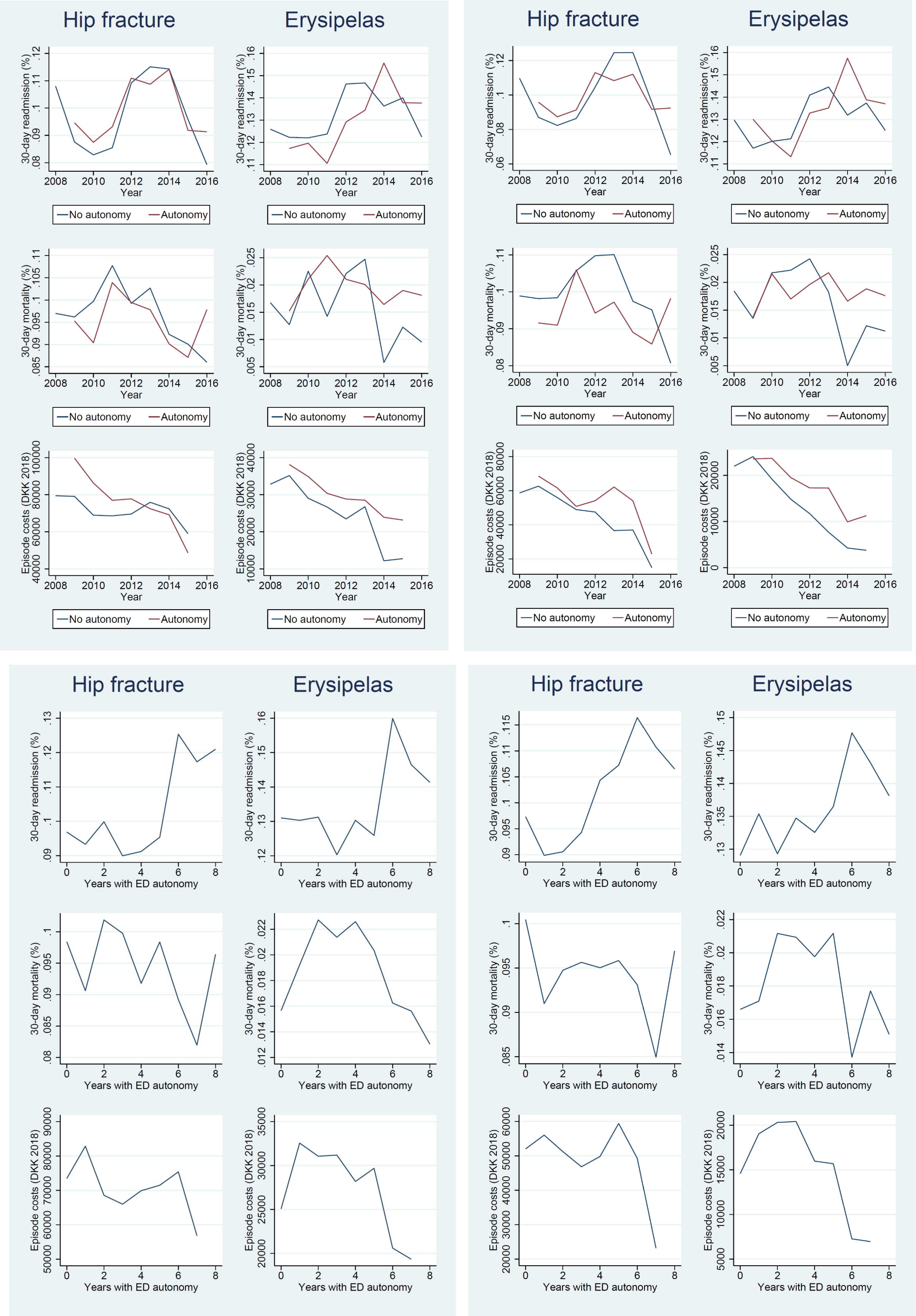

Supplement: S2 Fig — A. Unadjusted outcomes over time for episodes managed by departments with versus departments without increased autonomy. Note: Values are yearly means across departments. B. Adjusted outcomes over time for episodes managed by departments with versus departments without increased autonomy. Note: Values are yearly means across departments. Adjustment is based on the mixed effects models of the main analysis, which includes all variables shown in manuscript Table 2. C. Unadjusted outcomes over the duration of time with increased autonomy (time since implementation). Note: Values are yearly means across departments. D. Adjusted outcomes over the duration of time with increased autonomy (time since implementation). Note: Values are yearly means across departments. Adjustment is based on the mixed effects models of the main analysis, which includes all variables shown in manuscript Table 2. (TIF) [file pone.0283325.s002.tif]
