# Supplementary material for: Emergency department reorganisation introducing increased autonomy: A mixed effects approach to evaluate the effects of a national policy
Source: PLoS One. 2023 Mar 23;18(3):e0283325. doi: 10.1371/journal.pone.0283325 (PMC10035920; doi:10.1371/journal.pone.0283325)
Supplement: S1 Table — ED = Emergency department, EPI = episode, DPT = Department. (DOCX) [file pone.0283325.s003.docx]

|  | **Variables** | **Scaling** | **Time of observation** |
| --- | --- | --- | --- |
| **Outcome** | 30-day readmission | Dummy | Episode year |
|  | 30-day mortality | Dummy | Episode year |
|  | Episode cost (DKK 2018) | Continuous | Episode year |
| **Intervention** | Duration of senior physician´s employment at the ED (year) | Continuous | Episode year |
| **Time** | Admission date (year) | Dummy | Episode year |
| **Department-level heterogeneity** | Teaching hospital | Dummy | Episode year – 1 |
|  | Annual episode volume (number) | Continuous | Episode year – 1 |
|  | Annual mean 30-day mortality (%) | Continuous | Episode year – 1 |
|  | Annual mean 30-day readmission (%) | Continuous | Episode year – 1 |
|  | Annual mean episode cost (DKK 2018) | Continuous | Episode year – 1 |
| **Episode-level heterogeneity** | Male gender | Dummy | - |
|  | Age (year) | Continuous | Episode year |
|  | Elixhauser Co-morbidity Index | 31 dummies | Episode year |
