# Supplementary material for: Emergency department reorganisation introducing increased autonomy: A mixed effects approach to evaluate the effects of a national policy
Source: PLoS One. 2023 Mar 23;18(3):e0283325. doi: 10.1371/journal.pone.0283325 (PMC10035920; doi:10.1371/journal.pone.0283325)
Supplement: S1 Questionnaire — (DOCX) [file pone.0283325.s004.docx]

**Questionnaire**

English version

How many years have you been employed in your current position at the emergency department?

(0) ❑ <1

(1) ❑ 1

(2) ❑ 2

(3) ❑ 3

(4) ❑ 4

(5) ❑ 5

(6) ❑ 6

(7) ❑ 7

(8) ❑ 8

(9) ❑ 9

(10) ❑ 10

(11) ❑ >10

In how many buildings are the emergency department situated?

(3) ❑ In one building

(4) ❑ In two buildings

(5) ❑ In three buildings

(6) ❑ In four buildings

(7) ❑ In more than four buildings

(8) ❑ I do not know

From which year do you think the above statement applies?

(1) ❑ Before 2008

(2) ❑ 2008

(3) ❑ 2009

(4) ❑ 2010

(5) ❑ 2011

(6) ❑ 2012

(7) ❑ 2013

(8) ❑ 2014

(9) ❑ 2015

(10) ❑ 2016

(13) ❑ 2017

(11) ❑ I do not know

(12) ❑ Not relevant

Is there normally senior physicians present day and night at the emergency department?

(1) ❑ Yes

(2) ❑ No

(3) ❑ I do not know

**From which year do you think the above statement applies?**

(1) ❑ Before 2008

(2) ❑ 2008

(3) ❑ 2009

(4) ❑ 2010

(5) ❑ 2011

(6) ❑ 2012

(7) ❑ 2013

(8) ❑ 2014

(9) ❑ 2015

(10) ❑ 2016

(13) ❑ 2017

(11) ❑ I do not know

(12) ❑ Not relevant

**Are senior physicians employed at the emergency department?**

(1) ❑ Yes

(2) ❑ No

(3) ❑ I do not know

From which year do you think the above statement applies?

(1) ❑ Before 2008

(2) ❑ 2008

(3) ❑ 2009

(4) ❑ 2010

(5) ❑ 2011

(6) ❑ 2012

(7) ❑ 2013

(8) ❑ 2014

(9) ❑ 2015

(10) ❑ 2016

(13) ❑ 2017

(11) ❑ I do not know

(12) ❑ Not relevant

How are the senior physicians from the other hospital departments at present affiliated to the emergency department?

(1) ❑ They are being send for when needed

(2) ❑ They are available for conference calls related to their medical specialty

(3) ❑ They are send for to receive their own patients (related to their specialty) in the emergency department

(4) ❑ They take shifts at the emergency department as part of their regular schedule

(5) ❑ I do not know

From which year do you think the above statement applies?

(1) ❑ Before 2008

(2) ❑ 2008

(3) ❑ 2009

(4) ❑ 2010

(5) ❑ 2011

(6) ❑ 2012

(7) ❑ 2013

(8) ❑ 2014

(9) ❑ 2015

(10) ❑ 2016

(13) ❑ 2017

(11) ❑ I do not know

(12) ❑ Not relevant

Do you have a flow coordinator (a physician or nurse who is responsible for creating patient flow in the emergency department)?

(1) ❑ Yes

(2) ❑ No

(3) ❑ I do not know

From which year do you think the above statement applies?

(1) ❑ Before 2008

(2) ❑ 2008

(3) ❑ 2009

(4) ❑ 2010

(5) ❑ 2011

(6) ❑ 2012

(7) ❑ 2013

(8) ❑ 2014

(9) ❑ 2015

(10) ❑ 2016

(13) ❑ 2017

(11) ❑ I do not know

(12) ❑ Not relevant

Do you have multidisciplinary teams, who are cooperating and discussing diagnostic and treatment options for the patients?

(1) ❑ Yes

(2) ❑ No

(3) ❑ I do not know

From which year do you think the above statement applies?

(1) ❑ Before 2008

(2) ❑ 2008

(3) ❑ 2009

(4) ❑ 2010

(5) ❑ 2011

(6) ❑ 2012

(7) ❑ 2013

(8) ❑ 2014

(9) ❑ 2015

(10) ❑ 2016

(13) ❑ 2017

(11) ❑ I do not know

(12) ❑ Not relevant

To what extent is it possible for the senior physicians to make decisions about patient treatment without consulting physician from other departments?

(1) ❑ Never

(2) ❑ To a very small extent

(3) ❑ To a small extent

(4) ❑ To a moderat extent

(5) ❑ To a great extent

(6) ❑ To a very great extent

(7) ❑ Always

(8) ❑ I do not know

From which year do you think the above statement applies?

(1) ❑ Before 2008

(2) ❑ 2008

(3) ❑ 2009

(4) ❑ 2010

(5) ❑ 2011

(6) ❑ 2012

(7) ❑ 2013

(8) ❑ 2014

(9) ❑ 2015

(10) ❑ 2016

(13) ❑ 2017

(11) ❑ I do not know

(12) ❑ Not relevant

Danish version

Hvor mange år har du været ansat i din nuværende stilling i akutafdelingen?

(0) ❑ <1

(1) ❑ 1

(2) ❑ 2

(3) ❑ 3

(4) ❑ 4

(5) ❑ 5

(6) ❑ 6

(7) ❑ 7

(8) ❑ 8

(9) ❑ 9

(10) ❑ 10

(11) ❑ >10

I hvor mange bygninger er akutafdelingens forskellige afsnit placeret?

(3) ❑ I en bygning

(4) ❑ I to bygninger

(5) ❑ I tre bygninger

(6) ❑ I fire bygninger

(7) ❑ I mere end fire bygninger

(8) ❑ Ved ikke

Fra hvilket årstal vil du mene, at denne placering har været gældende?

(1) ❑ Før 2008

(2) ❑ 2008

(3) ❑ 2009

(4) ❑ 2010

(5) ❑ 2011

(6) ❑ 2012

(7) ❑ 2013

(8) ❑ 2014

(9) ❑ 2015

(10) ❑ 2016

(13) ❑ 2017

(11) ❑ Ved ikke

(12) ❑ Ikke relevant

Er der i dag som udgangspunkt speciallæger til stede i akutafdelingen i døgndækning?

(1) ❑ Ja

(2) ❑ Nej

(3) ❑ Ved ikke

Fra hvilket årstal vil du mene, at ovenstående besvarelse har været gældende?

(1) ❑ Før 2008

(2) ❑ 2008

(3) ❑ 2009

(4) ❑ 2010

(5) ❑ 2011

(6) ❑ 2012

(7) ❑ 2013

(8) ❑ 2014

(9) ❑ 2015

(10) ❑ 2016

(13) ❑ 2017

(11) ❑ Ved ikke

(12) ❑ Ikke relevant

**Er der i dag ansat speciallæger i akutafdelingen?**

(1) ❑ Yes

(2) ❑ No

(3) ❑ I do not know

Fra hvilket årstal vil du mene, at ovenstående besvarelse har været gældende?

(1) ❑ Før 2008

(2) ❑ 2008

(3) ❑ 2009

(4) ❑ 2010

(5) ❑ 2011

(6) ❑ 2012

(7) ❑ 2013

(8) ❑ 2014

(9) ❑ 2015

(10) ❑ 2016

(13) ❑ 2017

(11) ❑ Ved ikke

(12) ❑ Ikke relevant

På hvilken måde er speciallægerne fra sygehusets andre afdelinger primært tilknyttet akutafdelingen i dag?

(1) ❑ De kan tilkaldes ved behov

(2) ❑ De er tilgængelige for telefonkonference relateret til deres speciale

(3) ❑ De tilkaldes til at modtage egne specialepatienter i akutafdeling

(4) ❑ De tager vagter på skift i akutafdelingen som en fast del af arbejdsplanen

(5) ❑ Ved ikke

Fra hvilket årstal vil du mene, at denne tilknytning har været gældende?

(1) ❑ Før 2008

(2) ❑ 2008

(3) ❑ 2009

(4) ❑ 2010

(5) ❑ 2011

(6) ❑ 2012

(7) ❑ 2013

(8) ❑ 2014

(9) ❑ 2015

(10) ❑ 2016

(13) ❑ 2017

(11) ❑ Ved ikke

(12) ❑ Ikke relevant

Anvender I en flowkoordinator (en læge eller sygeplejerske der er ansvarlig for at skabe flow i akutafdelingen)?

(1) ❑ Ja

(2) ❑ Nej

(3) ❑ Ved ikke

Fra hvilket årstal vil du mene, at dette har været gældende?

(1) ❑ Før 2008

(2) ❑ 2008

(3) ❑ 2009

(4) ❑ 2010

(5) ❑ 2011

(6) ❑ 2012

(7) ❑ 2013

(8) ❑ 2014

(9) ❑ 2015

(10) ❑ 2016

(13) ❑ 2017

(11) ❑ Ved ikke

(12) ❑ Ikke relevant

Anvender I tværfaglige teams, der løbende samarbejder og diskuterer diagnosticering og behandling af patienterne?

(1) ❑ Ja

(2) ❑ Nej

(3) ❑ Ved ikke

Fra hvilket årstal vil du mene, at dette har været gældende?

(1) ❑ Før 2008

(2) ❑ 2008

(3) ❑ 2009

(4) ❑ 2010

(5) ❑ 2011

(6) ❑ 2012

(7) ❑ 2013

(8) ❑ 2014

(9) ❑ 2015

(10) ❑ 2016

(13) ❑ 2017

(11) ❑ Ved ikke

(12) ❑ Ikke relevant

I hvilket omfang kan speciallæger i akutafdelingen træffe beslutninger om behandling uden at konsultere læger fra andre afdelinger?

(1) ❑ Aldrig

(2) ❑ I meget lille omfang

(3) ❑ I mindre omfang

(4) ❑ I moderat omfang

(5) ❑ I stort omfang

(6) ❑ I meget stort omfang

(7) ❑ Altid

(8) ❑ Ved ikke

Fra hvilket årstal vil du mene, at det angivne omfang har været gældende?

(1) ❑ Før 2008

(2) ❑ 2008

(3) ❑ 2009

(4) ❑ 2010

(5) ❑ 2011

(6) ❑ 2012

(7) ❑ 2013

(8) ❑ 2014

(9) ❑ 2015

(10) ❑ 2016

(13) ❑ 2017

(11) ❑ Ved ikke

(12) ❑ Ikke relevant
